# Hematogenous Long Bone Osteomyelitis by *Prevotella (Bacteroides) Melaninogenicus*

**DOI:** 10.4021/jocmr465w

**Published:** 2010-12-11

**Authors:** Panagiotis K Karabinas, Evangelopoulos Dimitrios Stergios, Marina G Athanasopoulou, John Vlamis

**Affiliations:** a 1st Orthopedic Department, University of Athens, Greece; b 3rd Orthopedic Department, University of Athens, Greece; c Department of Biopathology, 7th I.K.A Hospital, Athens, Greece

## Abstract

Anaerobic infections are difficult to recognize and may lead to life threatening complications. We present the case of an acute *Prevotella* hematogenous infection of the humerus in a previously healthy adult patient, treated with a two-stage surgical treatment, emphasizing on the importance of the radical surgical debridement and the selection of the appropriate antimicrobial agents. To our knowledge, such an acute hematogenous infection has not been previously reported in the literature.

**Keywords:**

Osteomyelitis; Gram-negative hematogenous infections; Two stage surgical treatment

## Introduction

Recent studies using improved methods of isolation and culture report that anaerobes account for 33% of all organisms responsible for osteomyelitis [1, 2]. The main causes of acute osseous hematogenous infections in adults are *Staphylococcus aureus* and occasionally *Enterobacter* or *Streptococci* [1-3]. Factors predisposing to bone infection include vascular disease, bites, contiguous infections, peripheral neuropathies, diabetes mellitus, decubitus ulcers, and trauma. There are specific methods for the collection, transportation and culture of these species [4, 5]. Treatment of such infections is often complicated either due to their resistance to antimicrobial agents (produce the enzyme beta-lactamase) or/and to the polymicrobial synergy of these infections. Treated early and appropriately, the disease responds rapidly [6-8].

*Prevotella* (pigmented) *melaninogenica* and (non-pigmented) *oralis* are anaerobic gram-negative bacilli belonging to the *Bacteroides* species [9]. These organisms represent predominant components of the normal bacterial florae of the mucous membranes (oral and vaginal) and may be the cause of several endogenous infections. They have been detected from infections of the central nervous system, neck, chest, abdomen, pelvis and skin [5, 8, 10-13]. Rarely, these organisms can be isolated from joint and bone infections (metaphysis of the long bones and skull) causing acute or chronic septic arthritis and osteomyelitis. Nevertheless, these infections are considered to be secondary to contiguous sites of infection and rarely follow bacteremia [8, 9].

## Case Report

A 35-year-old previously healthy male presented to our Emergency Department with a 4-day history of fever (38.5^o^C), progressive swelling, and pain at the distal third of the humerus. After thorough clinical, physical and laboratory examination [c-reactive protein (CRP), erythrocyte sedimentation (ESR) and Leukocyte count], a local infection was diagnosed.

**Figure 1.**
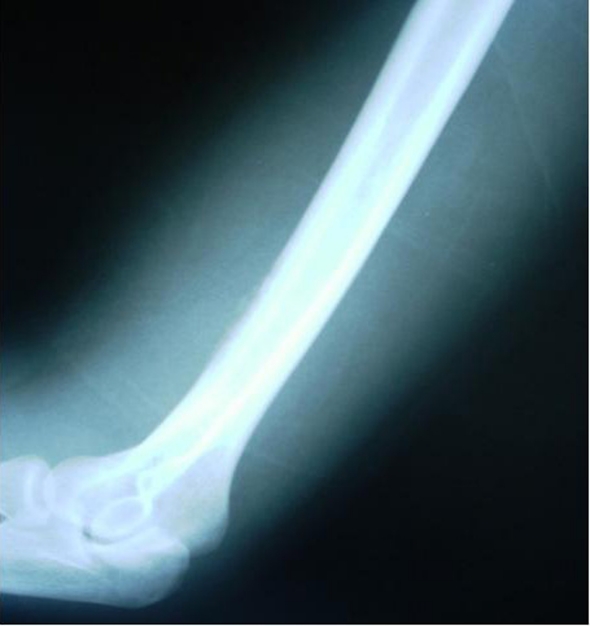
Lateral x-ray demonstrating the osseous reaction at the distal third of the humerus at the time of admission.

The radiological evaluation confirmed the suspicion of an acute osteomyelitis demonstrating increased inflammatory osseous reaction (Fig. 1). However, the route of infection was unclear. Despite the thorough clinical and radiological investigation, no contiguous infection could be detected and no predisposing factors were reported.

The patient was submitted to surgical draining of the soft tissue pus, debridement of the necrotic tissue and the abscesses, as well as bone decompression to improve circulation and increase tissue oxygenation. The pus and the debrided tissues were sent to the lab for anaerobic and aerobic cultivation. The results revealed an anaerobic infection caused by *Prevotella* pigmented *Melaninogenicus* and non-pigmented *Oralis*. Proper antibiotic treatment was defined according to the antibiogram and was administered intravenously. A week later the patient was discharged from our clinic with the appropriate per os antibiotic treatment.

**Figure 2.**
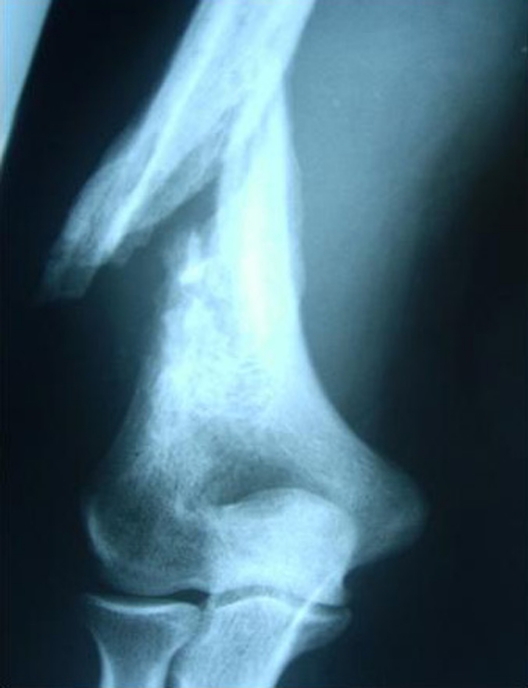
AP x-ray of the elbow joint demonstrating the fracture of the distal third of the humerus at the site of bone decompression.

Ten days later, he visited the outpatient clinic complaining for intense pain at the distal third of the humerus after a violent movement of his arm. The radiological examination revealed a humeral fracture at the area of bone decompression (Fig. 2).

**Figure 3.**
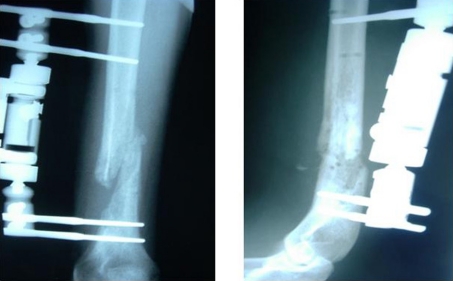
AP and lateral x-rays demonstrating the stabilization of the fracture of the distal humerus by means of a monolateral external fixator.

Initially, the fracture was stabilized with a monolateral external fixator (Orthofix) and the bone was once more decompressed (Fig. 3). The ex-fix was kept in place for a period of two months and proper antibiotics were administered until CRP and ESR values returned to normal.

**Figure 4.**
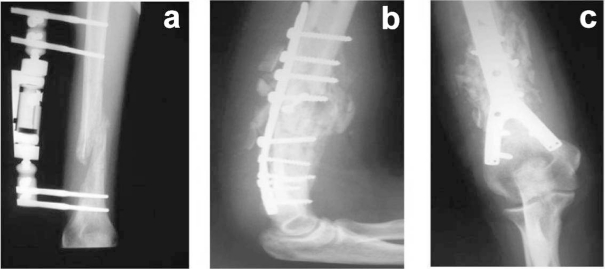
(a) AP x-ray demonstrating the absence of callus formation; (b, c) Postoperative AP and lateral x-rays after osteosynthesis of the fracture by means of a 'Y' plate non-union of the fracture after the end of antibiotic therapy and internal fixation of the non-union of the humerus.

Two months after surgery, since no signs of callus formation were observed at the fracture area, our patient was submitted to a second surgery for internal fixation of the fracture by means of a 'Y' plate (Fig. 4). At that time, all laboratory values (CRP, ESR, and tissue and material cultures) were negative and there were no clinical and radiological signs of infection.

**Figure 5.**
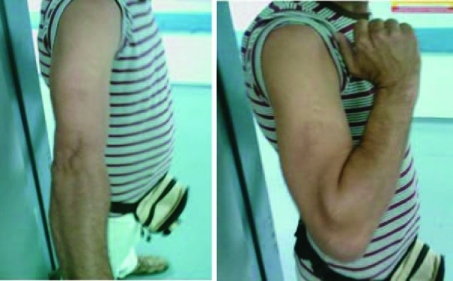
Elbow joint, flexion-extension: full range of motion.

Three months after the second surgery, clinical examination revealed the absence of pain and full range of motion of the elbow. The radiological evaluation demonstrated fracture healing and all serological markers (ESR, CRP) remained within normal range (Fig. 5).

## Discussion

The *Prevotella (Bacteroides* species) are part of the normal oral gastrointestinal and genital tract flora. They are often isolated from mixed infections with other anaerobes and aerobic or facultative species.

Laboratory findings, such as high ESR, can support the initial clinical suspicion. Radiological and imaging studies may assist the diagnosis, revealing the presence of gas in the infected site. On the other hand, normal Tc-99 MDP and gallium images virtually exclude the diagnosis of an infection. MRI with its high specificity and sensitivity may serve as an important diagnostic tool [1, 2, 9]. Due to the early recognition and administration of proper antimicrobial therapy, the mortality rate (1%) has decreased significantly [8]. Other than long bones, vertebral osteomyelitis is also infrequent, accounting for only 2-4% of osteomyelitis in adults [1, 2].

The treatment efficacy for such serious infections depends on three basic principles: (1) the control and neutralization of the produced toxins, (2) the control of tissue environment to prevent local bacterial proliferation and (3) the limitation of bacterial spread. In the few cases of vertebral osteomyelitis by *Prevotella (Bacteroides) Melaninogenicus* reported in the literature, an osseous biopsy and thorough lab analysis of the biopsy specimens, by means of cultures for both aerobic and anaerobic specimens, were mandatory [1, 2]. Additionally, the accurate and thorough debridement of necrotic tissues as well as bone decompression, for improvement of circulation and tissue oxygenation, are of outmost importance. Antimicrobial therapy should always be combined with appropriate surgical procedures and must be focused on both aerobic and anaerobic organisms. Administered antibiotics must be selected according to the performed antibiogram [8, 9].

The ability of these organisms to induce abscess formation is correlated with the presence of a mucopolysaccharide capsule, suppressessing phagocytosis [12, 13]. The last two decades these anaerobic bacteria demonstrate resistance to penicillin through production of β-lactamase. The addition of a β-lactamase inhibitor to penicillin can make standard treatment more efficient. Other effective antimicrobic agents include clindamycin, metronidazole, cefoxitin, chloramphenicol and imipenem [8, 9].

## Conclusions

To our knowledge, a case of an acute *Prevotella* hematogenous infection at the metaphysis of a long bone, in a previously healthy male, has never been reported in the literature. In case of long bone osteomyelitis the surgical therapy is of critical importance. It must include surgical draining of the abscesses, thorough debridement of the necrotic tissues, as well as osseous decompression (bone drillings, bone fenestration). Moreover, it is essential to provide a mechanical stability of the decompressed bone when required. In a first stage, fixation can be accomplished by means of an external fixator, thus providing temporary stability until clinical signs of inflammation subside and laboratory results return to normal. It is of great importance to maintain the length of the infected bone as well as the range of motion of the proximal and distal joints. Treated early and appropriately, the disease responds rapidly. In a second stage, when healing of the soft tissue envelope and sterilization of the bone are achieved, a rigid fixation (internal fixation) can be performed if required, to enhance bone regeneration. We believe that the two-stage approach to osteomyelitis of long bones is a safe approach providing good long-term results.
